# CD79A and GADD45A as novel immune-related biomarkers for respiratory syncytial virus severity in children: an integrated machine learning analysis and clinical validation

**DOI:** 10.3389/fimmu.2025.1609183

**Published:** 2025-07-03

**Authors:** Juan Juan Chen, Zhang Ze Lu, Yu Xin Jing, Xing Mei Nong, Yi Qin, Jin Yang Huang, Na Lin, Jie Wei

**Affiliations:** ^1^ Department of Pediatrics, Affiliated Hospital of Youjiang Medical University for Nationalities, Baise, Guangxi, China; ^2^ Department of Urology, Baise People’s Hospital, Baise, Guangxi, China; ^3^ Department of Hematology, The First Affiliated Hospital of Guangxi Medical University, Nanning, Guangxi, China

**Keywords:** RSV, biomarkers, CD79A and GADD45A, machine learning, severity assessment

## Abstract

**Background:**

Respiratory syncytial virus (RSV) is a leading cause of severe lower respiratory infections in children, yet biomarkers for assessing disease severity remain limited. Herein, we investigated the differential expression biomarkers between RSV infected hospitalized patients, healthy groups and RSV infected outpatients.

**Methods:**

Two publicly available transcriptomic datasets (GSE77087 and GSE188427) were retrieved from the Gene Expression Omnibus (GEO) database. The GSE77087 dataset comprised peripheral blood samples from 81 children with confirmed RSV infection (61 hospitalized and 20 outpatient) and 23 healthy controls. The GSE188427 dataset included 147 RSV-infected children (113 hospitalized and 34 outpatient) and 51 healthy controls. Genes with |log2 fold change (logFC)| > 0 and false discovery rate (FDR) < 0.05 were considered differentially expressed. Overlapping DEGs between the two datasets were identified using the VennDiagram package. Gene Ontology (GO) and Kyoto Encyclopedia of Genes and Genomes (KEGG) pathway enrichment analyses were conducted on the intersecting DEGs via the clusterProfiler package, with terms deemed significant at FDR < 0.05.The CIBERSORT algorithm was applied to estimate the relative proportions of 22 immune cell types in 228 RSV-infected samples. Potential drug interactions for hug genes were predicted using the Drug-Gene Interaction Database (DGIdb). Competing endogenous RNA (ceRNA) networks were constructed using the SpongeScan database to identify lncRNAs interacting with the target miRNAs. Networks were visualized using Cytoscape (v3.10.1).Finally, Machine Learning-Based Biomarker Selection and hub gene identification and validation

**Results:**

Differential gene expression analysis revealed 81 overlapping genes between hospitalized and outpatient RSV-infected children. Machine learning models, particularly SVM (area under the curve, AUC = 0.950), prioritized CD79A and GADD45A as key predictors of hospitalization. CD79A was significantly downregulated in severe cases, correlating with impaired B-cell responses and cytotoxic immunity, while GADD45A, upregulated in severe infections, linked to oxidative stress and neutrophil-driven inflammation. Immune cell profiling highlighted distinct infiltration patterns, with severe cases showing elevated naïve B cells and M0 macrophages versus activated NK cells and M1 macrophages in mild cases. Clinical validation in 92 children confirmed CD79A suppression and GADD45A elevation in severe RSV (p < 0.001), aligning with younger age, lower weight, and respiratory distress. Functional enrichment implicated endoplasmic reticulum stress and neutrophil extracellular traps in disease progression. Drug-target predictions and ceRNA networks further revealed therapeutic potential.

**Conclusion:**

These findings establish CD79A and GADD45A as clinically actionable biomarkers for RSV severity, offering insights into immune dysregulation and guiding personalized management strategies.

## 1 Introduction

Human respiratory syncytial virus (RSV) is an enveloped, negative-strand RNA virus belonging to the Pneumoviridae family of the Mononegavirales order. RSV is a ubiquitous pathogen that affects people of all age groups, with a significant impact on the pediatric population. children, immunocompromised individuals, and the elderly are at risk of developing severe RSV infections. RSV exhibits a seasonal transmission pattern, typically peaking during the colder months in temperate regions, where RSV infections often reach their apex. RSV is a leading cause of severe acute lower respiratory infections (ALRI) among children worldwide (1). Bronchiolitis, primarily caused by various respiratory viruses and most commonly by RSV, is a major reason for children hospitalization (2). It is estimated that RSV causes approximately 100,000 deaths among children under 5 years old annually, with the highest death toll in developing countries (3). RSV also contributes to a significant disease burden among young children. In 2015, it was estimated that 3.2 million hospitalizations in this age group were due to RSV (4). Additionally, approximately 500,000 hospitalizations and deaths were attributed to RSV-related acute respiratory infections among the elderly in 2015 (5). The only method to prevent bronchiolitis is palivizumab, a monoclonal antibody targeting the RSV virus. However, this medication is expensive, costing between $3,221 and $12,568 for protection during the entire RSV season (6). The percentage of pediatric intensive care unit(PICU)/high dependency unit (HDU) admissions is 11.6% for RSV alone, compared to 10.6% for the RSV-negative group, depending on the children’s birth weight and time of birth (7).

Based on RSV leading to lots of the children and elder people suffer acute respiratory infection, it will be demonstrated the overall cost burden of RSV on health services, supporting the need for prevention and improved clinical management. The most important is the with the better assessing the severity of RSV infection, especially for children RSV infected patients.

Despite the clinical importance of RSV, current biomarkers for assessing disease severity remain limited. To date, previous study indicated that the innate immune system plays a crucial role in defending against RSV (8). Although some pattern recognition receptors (PRRs) in the innate immune system can sense RSV pathogen-associated molecular patterns (PAMPs) and induce interferon (IFN) production, nasal washes from RSV-infected children contain only low to undetectable levels of IFN-α and IFN-β (9, 10). This is unlike other respiratory viruses, such as influenza A virus and parainfluenza virus (11). Furthermore, monocytes from children with RSV-induced bronchiolitis produce very low levels of IFN-α (12). These finding indicated that the immune system response play the critical roles for RSV infection process. And the immune response can have a deep influence on the severity of RSV infection. However, these traditional biomarkers lack the specificity and sensitivity required to accurately predict disease progression and severity.

There is an urgent need to understand the immune system status of RSV-positive and RSV-negative children and young children. This understanding can help assess whether hospitalization is necessary and evaluate the differences in the immune systems of patients with mild and severe RSV infections. To address these limitations, our study employs an integrated multi-omics approach combined with machine learning algorithms. Unlike traditional methods that focus on individual biomarkers, machine learning can analyze complex datasets and identify patterns that are not immediately apparent. In this study, we first analyzed immune gene expression related to immune status in hospitalized and non-hospitalized RSV-positive children, as well as RSV-negative children, using public databases. Then, we constructed a model using machine learning to identify core differentially expressed molecular markers. Finally, we compared the clinical characteristics and test results of recent severe and non-severe RSV-infected children in our hospital and validated the expression levels of the identified immune status-related molecular markers. Our aim is to individualize treatment for pediatric RSV patients, reduce the economic and medical burden on families and society, and explore potential drug targets to improve the efficacy of RSV infection treatment.

## 2 Materials and methods

### 2.1 Data collection

We downloaded the GSE77087 dataset from the GEO database, which includes blood samples from 81 children diagnosed with respiratory syncytial virus (RSV) infection on the first day of diagnosis (20 outpatient and 61 inpatient) and 23 blood samples from children without RSV infection. Another dataset, GSE188427, contains blood samples from 147 children diagnosed with RSV infection on the first day of diagnosis (34 outpatient and 113 inpatient) and 51 blood samples from children without RSV infection.

We selected the GSE77087 and GSE188427 datasets based on the following considerations.

1. Both datasets are derived from peripheral blood samples with relatively large sample sizes, comprising 81 patients (61 hospitalized with severe RSV infection and 20 outpatient with mild symptoms) and 23 healthy controls in GSE77087, and 147 patients (113 hospitalized, 34 outpatient) and 51 healthy controls in GSE188427, which provides a solid statistical foundation;
2. GSE77087 was generated using the Illumina HumanHT-12 V4.0 expression beadchip platform (GPL10558), while GSE188427 was generated using the Affymetrix Clariom S Pico Assay HT platform (GPL25336). The use of distinct representative platforms facilitates validation of findings across platforms;
3. All patients were confirmed cases of RSV infection and were categorized into inpatient (severe) and outpatient (mild) groups. Peripheral blood samples were collected on the day of diagnosis, minimizing time-related variability.

Thus, these two datasets are complementary and suitable for identifying transcriptomic differences between severe and mild RSV infection in pediatric patients.

### 2.2 Differential gene expression analysis between inpatient and outpatient children

We used the ‘sva’ package (https://bioconductor.org/packages/release/bioc/html/sva.html ) in R (version 4.3.1) to perform batch correction and normalization independently for each dataset. Batch effects were removed using the ‘ComBat’ function (parameters batch = platform, mod = NULL), followed by z-score standardization of the combined expression matrix to ensure comparability across platforms. The resulting corrected expression matrix was used for downstream DEG identification and analyses. Differential genes expression (DEGs) analysis was conducted using the limma package (https://bioconductor.org/packages/release/bioc/html/limma.html), with a filtering criterion of absolute logFC > 0 and a false discovery rate (FDR) value < 0.05 to identify DEGs. The intersection of DEGs from the GSE77087 and GSE188427 datasets was obtained using the VennDiagram package for subsequent analysis.

### 2.3 Functional enrichment analysis of DEGs between inpatient and outpatient children

Gene Ontology (GO) and Kyoto Encyclopedia of Genes and Genomes (KEGG) functional enrichment analyses were performed on the DEGs between inpatient and outpatient children using the org.Hs.eg.db and clusterProfiler packages (https://bioconductor.org/packages/release/data/annotation/html/org.Hs.eg.db.html).

### 2.4 Machine learning-based feature gene selection for RSV

To obtain more information for predicting whether a child requires hospitalization, we combined the GSE77087 and GSE188427 datasets. We then applied machine learning algorithms, including Extreme Gradient Boosting (XGB), Random Forest (RF), Generalized Linear Models (GLM), and Support Vector Machines (SVM), using the insertSymbol, randomForest, xgboost, and kernlab packages to construct prediction models. Model optimization was based on the residual inverse cumulative distribution, minimum residual root mean square (RMSE), and receiver operating characteristic (ROC) curve area (AUC). Bar charts generated by the rms package were used to identify key genes in the prediction model. We selected the model with the smallest residual inverse cumulative distribution, the smallest RMSE, and the largest AUC for subsequent analysis.

### 2.5 Identification of hub RSV genes and their expression and diagnostic value

We performed differential gene expression analysis between RSV patients and normal controls in the GSE77087 and GSE188427 datasets. The intersection of the identified DEGs, the feature genes from the SVM model, and the DEGs between inpatient and outpatient children was used to obtain key hub genes. Data batch correction and merging were performed using the sva package, and expression levels of hub genes in normal, outpatient, and inpatient children were analyzed. We also used the pROC package to generate ROC curves to evaluate the diagnostic accuracy of determining the need for hospitalization based on the expression levels of hub genes.

### 2.6 Immune cell infiltration analysis in RSV between outpatient and inpatient children

To analyze immune cell differences between outpatient and inpatient children, we used the CIBERSORT algorithm (https://cibersort.stanford.edu/) to calculate the relative content of 22 immune cell types in 228 RSV patients. We also analyzed the correlation between the expression levels of hub genes and the extent of immune cell infiltration.

### 2.7 Potential drug targets and ceRNA network construction for hub genes

Potential drug targets for hub genes were predicted using the Drug-Gene Interaction Database (DGIdb) (https://www.dgidb.org/), and visualized using Cytoscape (version 3.10.1). We then used four databases (miRDB, miRanda, miRwalk, and TargetScan) to predict potential target miRNAs for the key genes. Only miRNAs identified in all four databases were considered candidate miRNAs. We subsequently used the SpongeScan database to predict lncRNAs associated with the target miRNAs. A ceRNA network consisting of mRNA-miRNA-lncRNA was constructed using Cytoscape (https://cytoscape.org/).

### 2.8 Validation analysis

We selected the sever RSV infected child patients and not severe RSV infected patients to compared the clinical characters, and collected their peripheral blood to perform PCR to explored the CD79A and GADD45A expression levels in RSV infected child patients and not severe RSV infected child patients. Total RNA was extracted from THP-1 and K562 cells using TRIzol reagent (Invitrogen). RNA integrity and quantity were confirmed by agarose gel electrophoresis and Nanodrop spectrophotometer (Thermo Fisher Scientific). High-quality RNA (A260/A280 ratio 1.8-2.0) was reverse-transcribed into cDNA using the PrimeScript RT reagent Kit (Takara Bio) following the manufacturer’s protocol. Primers for ER stress markers (HSP90AA1, ATF6, XBP1, IRE1, PERK, CHOP) and GAPDH were designed using Primer3 software and synthesized by Sangon Biotech. Primer sequences are listed in **Table 1**.qRT-PCR was performed using SYBR Green Real-time PCR Master Mix (Toyobo) on an ABI 7500 system (Applied Biosystems). The 20-µL reaction mixture included 10 µL SYBR Green Master Mix, 0.4 µL of each primer (10 µM), 2 µL cDNA template, and 7.2 µL RNase-free water. Thermal cycling 95°C for 30 s, followed by 40 cycles of 95°C for 5s and 60°C for 34s. Melting curve analysis verified product specificity. Relative gene expression levels were calculated using the 2^−ΔΔCt method, normalized to GAPDH. Each sample was run in triplicate, and experiments were repeated three times independently.

**Table 1:** Clinical baseline characteristics.

Parameter		Severe	General	X^2^	Z	P
Gender (n)	Male	18	39	0.072	—	0.788
	Female	12	23			
Term Children (n)	Yes	27	57	0.095	—	0.757
	No	3	5			
Dwelling environment (n)	Village	19	31	1.449	—	0.229
	City	11	31			
Background disease (n)	Yes	4	1	3.364	—	0.067
	No	26	61			
Pant (n)	Yes	14	9	11.146	—	0.001
	No	16	53			
Fever (n)	Yes	16	38	0.528	—	0.467
	No	14	24			
Increased C-reactive protein (n)	Yes	11	21	0.07	—	0.792
	No	19	41			
Age (years)	M (P25,P75)	0.4 (0.3, 0.8)	0.9 (0.6, 2.0)	—	3.9	<0.0001
Hospitalized weight (kg)	M (P25,P75)	7.35 (5.5, 9.0)	9.0 (7.5, 11.7)	—	3.78	<0.0001
Length of stay	M (P25,P75)	8.0 (7.0-10.3)	6.0 (5.0-6.0)	—	5.38	<0.0001

### 2.9 Statistical analysis

Continuous variables were expressed as median (interquartile range) and compared using the Mann-Whitney U test or Wilcoxon rank-sum test. Categorical variables were expressed as frequencies and percentages, and compared using the chi-square test or Fisher’s exact test. For differential gene expression analysis, the limma package was used to compute log2 fold changes, and p-values were adjusted using the Benjamini-Hochberg method (FDR <0.05 considered significant).Model performance was evaluated by receiver operating characteristic (ROC) curves and quantified by the area under the curve (AUC); comparisons of AUCs were performed using DeLong’s test. For immune cell proportion data from CIBERSORT, group comparisons were performed using Wilcoxon rank-sum tests, and correlations were assessed by Spearman’s rank correlation coefficient. All analyses were conducted in R version 4.3.1. Gene expression analyses utilized the limma, sva, and edgeR packages; machine learning algorithms were implemented using kernlab, randomForest, and xgboost; ROC analyses and DeLong tests were conducted using the pROC package. Data visualization was performed using ggplot2 and ComplexHeatmap. A two-sided p-value < 0.05 was considered statistically significant.

## 3 Results

### 3.1 Differential gene expression analysis between outpatient and inpatient children

**Figure 1:**
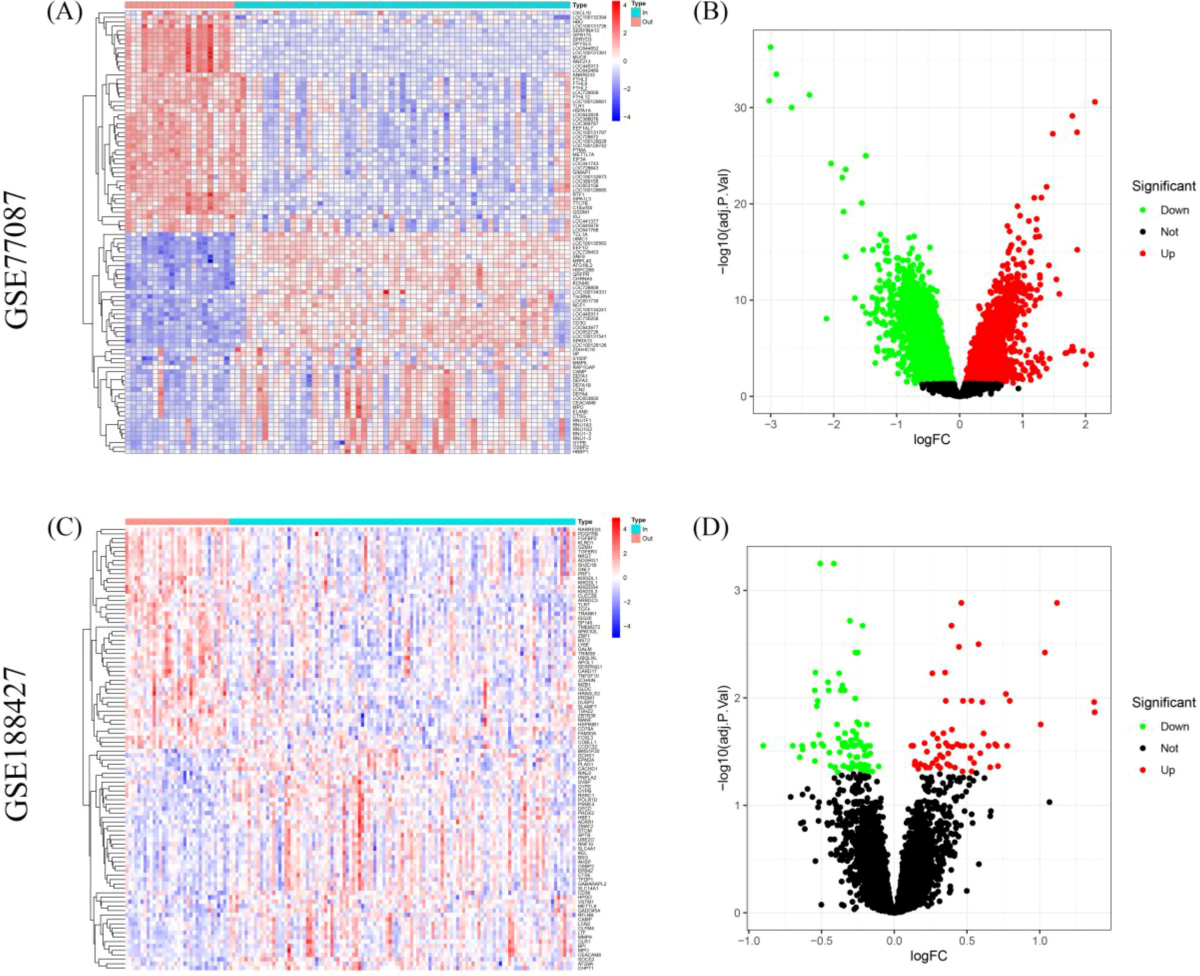
Differential gene expression analysis between outpatient and inpatient children with RSV. **(A, B)** Heatmap and volcano plots showing DEGs in the GSE77087 dataset between outpatient and inpatient children on the first day of diagnosis. **(C, D)** Heatmap and volcano plots for the GSE188427 dataset showing DEGs between outpatient and inpatient children.

Differential expression analysis revealed significant differences in gene expression between blood samples from outpatient and inpatient children on the first day of diagnosis in RSV-infected children. In the GSE77087 dataset, a total of 15,497 DEGs were identified, with 8,071 upregulated and 7,426 down-regulated genes (**Figures 1A, B**). In the GSE188427 dataset, 151 DEGs were identified, with 63 up-regulated and 88 downregulated genes (**Figures 1C, D**). This comparison was conducted to preliminary analyze potential molecular markers that differentiate severely ill children with RSV infection from those with mild RSV infection, and to explore whether there are markers that can distinguish disease severity.

### 3.2 Functional enrichment analysis of differential genes between outpatient and inpatient children

**Figure 2:**
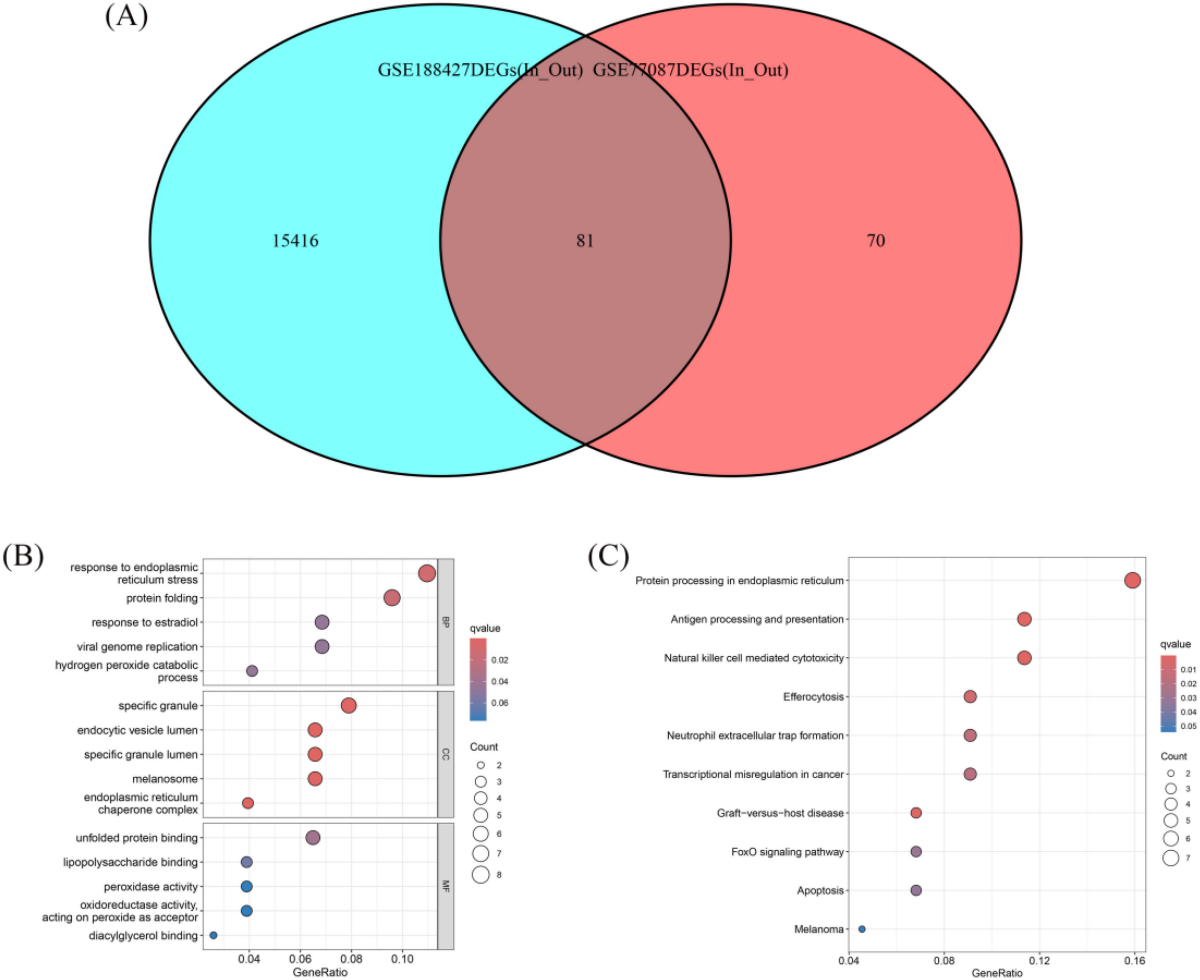
Venn diagram and functional enrichment of differential genes. **(A)** Venn diagram showing the overlap of DEGs between GSE77087 and GSE188427, identifying 81 common DEGs as potential biomarkers. **(B)** GO enrichment analysis of the 81 DEGs. **(C)** KEGG pathway analysis of the 81 DEGs.

Venn diagram analysis showed that the intersection of DEGs from the GSE77087 and GSE188427 datasets consisted of 81 DEGs, which were considered potential biomarkers for differentiating children requiring hospitalization (**Figure 2A**). GO and KEGG functional enrichment analyses of the 81 DEGs indicated that they were primarily involved in biological processes such as response to endoplasmic reticulum stress, protein folding, response to estradiol, viral genome replication, and hydrogen peroxide catabolic process. These genes were also enriched in cellular components including specific granules, endocytic vesicle lumen, specific granule lumen, melanosome, and endoplasmic reticulum chaperone complex. Additionally, they were associated with molecular functions such as unfolded protein binding, lipopolysaccharide binding, peroxidase activity, oxidoreductase activity acting on peroxide as acceptor, and diacylglycerol binding (**Figure 2B**). KEGG pathway analysis showed that these DEGs were involved in protein processing in endoplasmic reticulum, antigen processing and presentation, natural killer cell mediated cytotoxicity, efferocytosis, neutrophil extracellular trap formation, transcriptional misregulation in cancer, graft−versus−host disease, FoxO signaling pathway, apoptosis, and melanoma pathways (**Figure 2C**). These results highlight the significant role of RSV infection in immune regulation.

### 3.3 Machine learning-based feature gene selection for RSV

**Figure 3:**
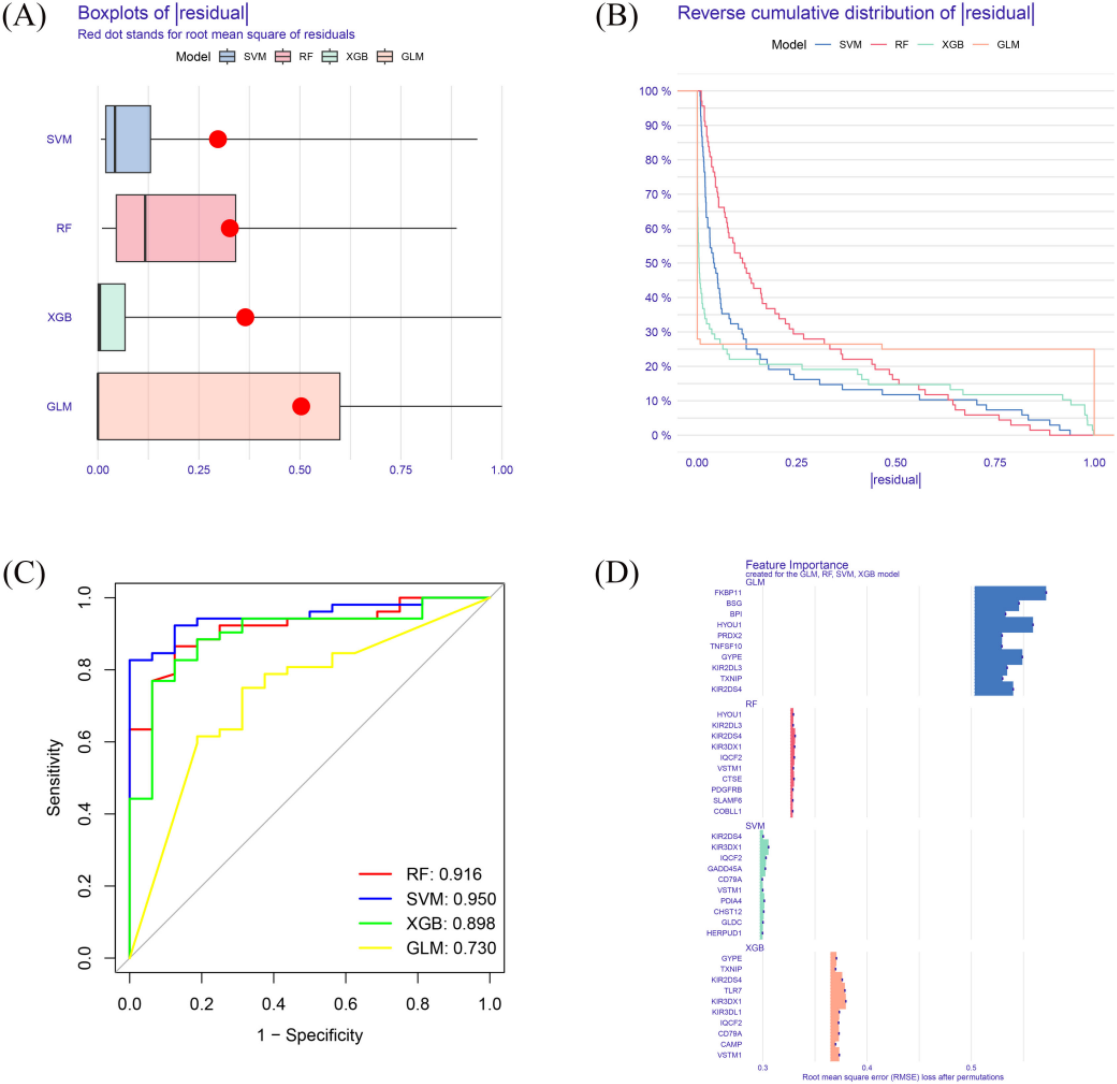
Machine learning model performance. **(A)** Residual RMSE for four machine learning models (XGB, RF, GLM, SVM). **(B)** Residual reverse cumulative distribution for each model. **(C)** ROC curve for each model. **(D)** key genes of high importance in each model.

After performing Venn analysis on the DEGs identified from GSE77087 and GSE188427, we selected the intersecting genes as candidate features. Subsequently, machine learning algorithms (XGBoost, RF, GLM, and SVM) were applied to prioritize key genes for predictive modeling. The SVM model was identified as the optimal model, based on the smallest RMSE (**Figure 3A**), smallest residual inverse cumulative distribution (**Figure 3B**), and the highest ROC curve AUC value (0.950) (**Figure 3C**), demonstrating high diagnostic accuracy. A total of 10 key genes were selected for further analysis, with high importance in the model (**Figure 3D**).

### 3.4 Identification and expression analysis of hub RSV genes

**Figure 4:**
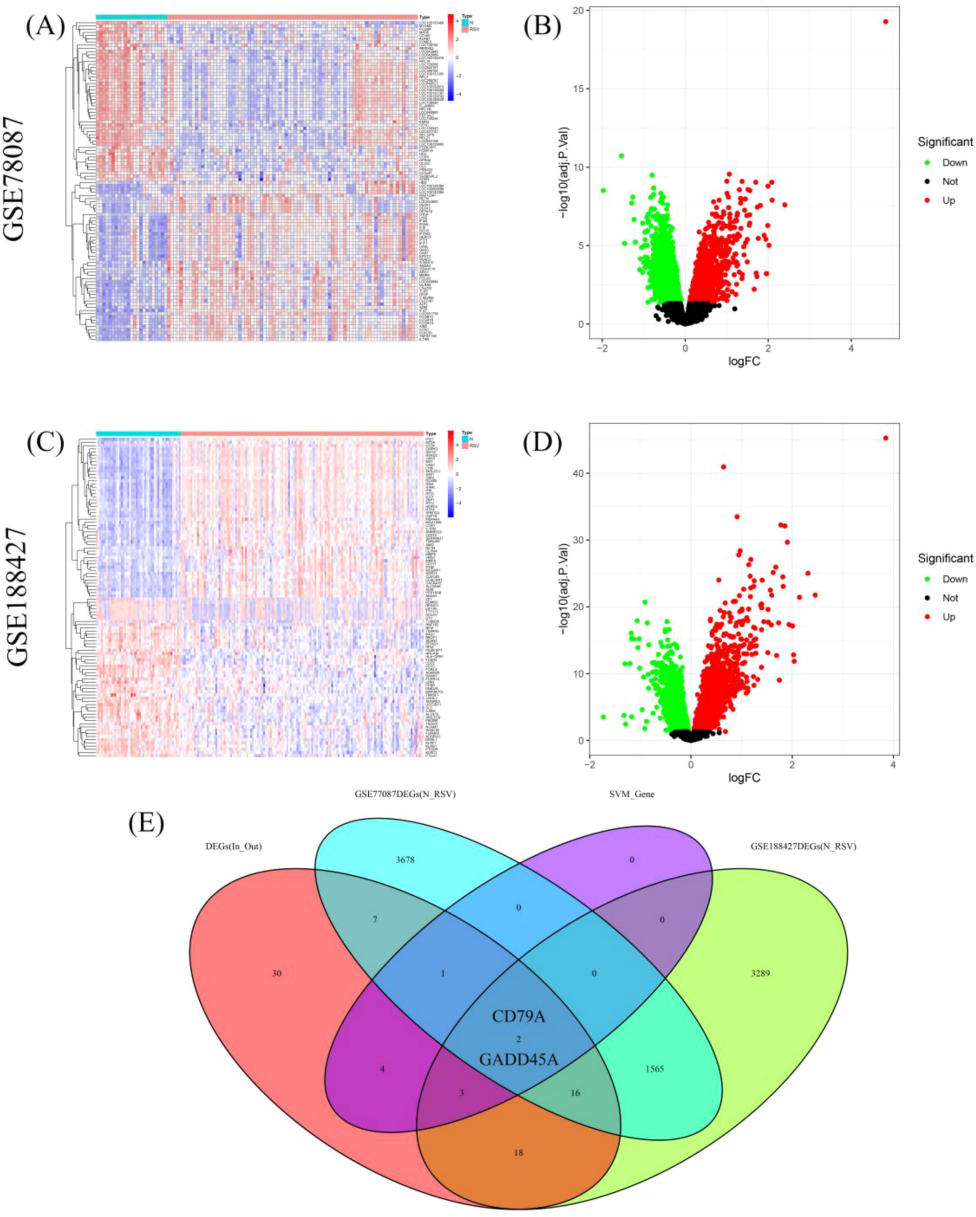
Hub genes identification. **(A, B)** Heatmap and volcano plots showing DEGs in GSE77087 between RSV-infected children and controls. **(C, D)** Heatmap and volcano plots showing DEGs in GSE188427 between RSV-infected children and controls. **(E)** Venn diagram showing hub genes identified from venn analyses: CD79A and GADD45A.

We first performed differential gene expression analysis between RSV-infected children and normal controls in the GSE77087 and GSE188427 datasets. In the GSE77087 dataset, a total of 5,269 DEGs were identified, with 2,153 upregulated and 3,116 downregulated genes (**Figures 4A, B**). In the GSE188427 dataset, 4,893 DEGs were identified, with 2,124 upregulated and 2,769 downregulated genes (**Figures 4C, D**). By intersecting the differential genes between normal and RSV-infected children, the differential genes between outpatient and inpatient children, and the feature genes from the SVM model, we identified two hub genes, CD79A and GADD45A (**Figure 4E**).

**Figure 5:**
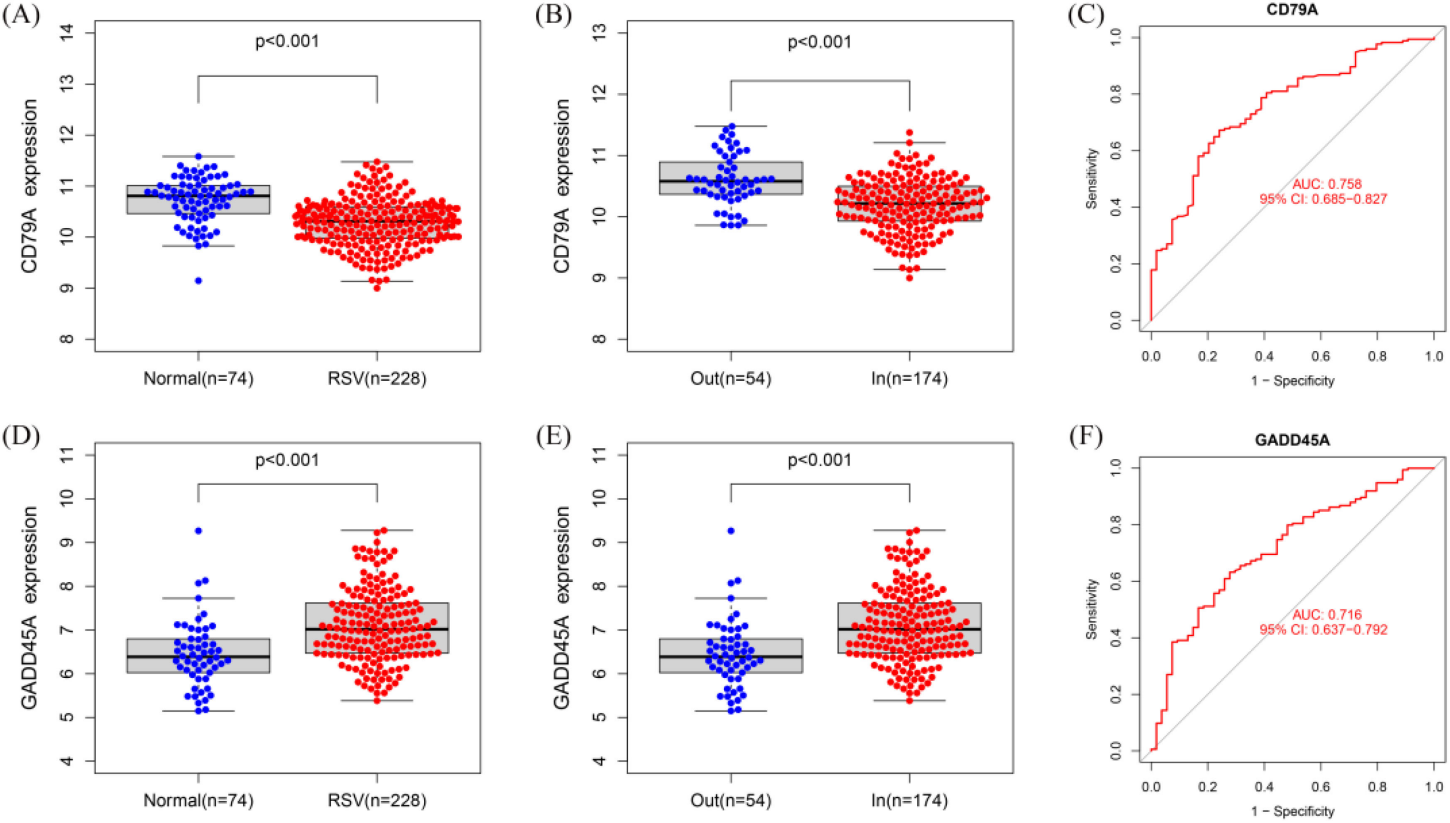
Expression and diagnostic value of hub genes. **(A, B)** Boxplot of CD79A expression levels in normal blood sample, RSV-infected outpatient children, and inpatient children. **(C)** ROC curve for CD79A in distinguishing inpatient from outpatient children (AUC = 0.758). **(D, E)** Boxplot of GADD45A expression levels in normal blood sample, RSV-infected outpatient children, and inpatient children expression. **(F)** ROC curve for GADD45A in distinguishing inpatient from outpatient children (AUC = 0.716).

Compared to normal control blood samples, CD79A was significantly downregulated in RSV-infected children, particularly in inpatient children (**Figures 5A, B**). ROC curve analysis demonstrated that the expression level of CD79A could effectively distinguish between inpatient and outpatient children, with an AUC value of 0.758 (**Figure 5C**).

In contrast, GADD45A was significantly upregulated in RSV-infected children, particularly in inpatient children (**Figures 5D, E**). ROC curve analysis showed that the expression level of GADD45A could also effectively differentiate between inpatient and outpatient children, with an AUC value of 0.716 (**Figure 5F**).

### 3.5 Immune cell infiltration analysis

**Figure 6:**
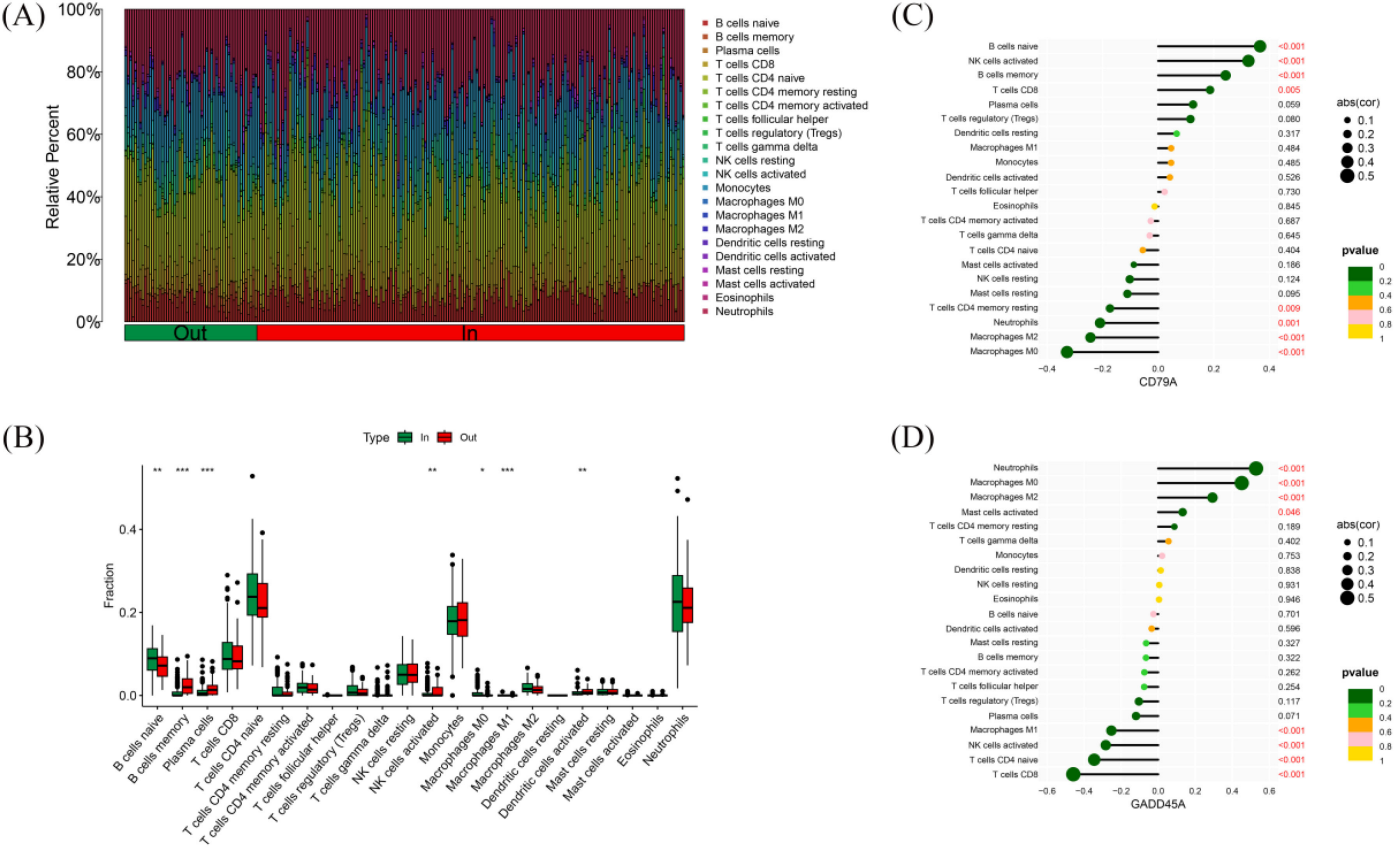
Immune cell infiltration in RSV-infected children. **(A)** Relative abundance of 22 immune cell types in 228 RSV-infected children. **(B)** Comparison of immune cell infiltration between outpatient and inpatient children. **(C)** Correlation between CD79A expression and immune cell infiltration. **(D)** Correlation between GADD45A expression and immune cell infiltration. * indicates p < 0.05, ** indicates p < 0.01, and *** indicates p < 0.001.

To ensure compatibility across platforms for immune cell infiltration analysis, we first performed batch correction. Then, the log2-RMA normalized values (from Affymetrix microarray, GSE77087) and the log2(CPM+1) values (from RNA-seq, GSE188427) were converted back to linear scale (i.e., 2^expression or CPM+1). The combined expression matrix was then standardized using z-score normalization and submitted as input to the CIBERSORT algorithm. CIBERSORT requires non-log-transformed expression data, and we ensured compliance with this requirement during preprocessing. Using the CIBERSORT algorithm, we calculated the relative immune cell infiltration of 228 RSV-infected children (**Figure 6A**). The results showed that the immune cell infiltration profiles of inpatient children were significantly different from those of outpatient children. In inpatient children, the relative content of naïve B cells and M0 macrophages was higher, while outpatient children showed higher levels of memory B cells, activated plasma cells, activated NK cells, M1 macrophages, and activated dendritic cells (**Figure 6B**). Correlation analysis between hub gene expression and immune cell infiltration levels showed that CD79A expression positively correlated with the infiltration of naïve B cells, activated NK cells, memory B cells, and CD8+ T cells, while negatively correlating with the infiltration of resting CD4+ T cells, neutrophils, M2 macrophages, and M0 macrophages (**Figure 6C**). GADD45A expression positively correlated with neutrophil, M0 macrophage, M2 macrophage, and activated mast cell infiltration, and negatively correlated with M1 macrophages, activated NK cells, naïve CD4+ T cells, and CD8+ T cell infiltration (**Figure 6D**).

### 3.6 Potential drug targets and ceRNA network construction for hub genes

**Figure 7:**
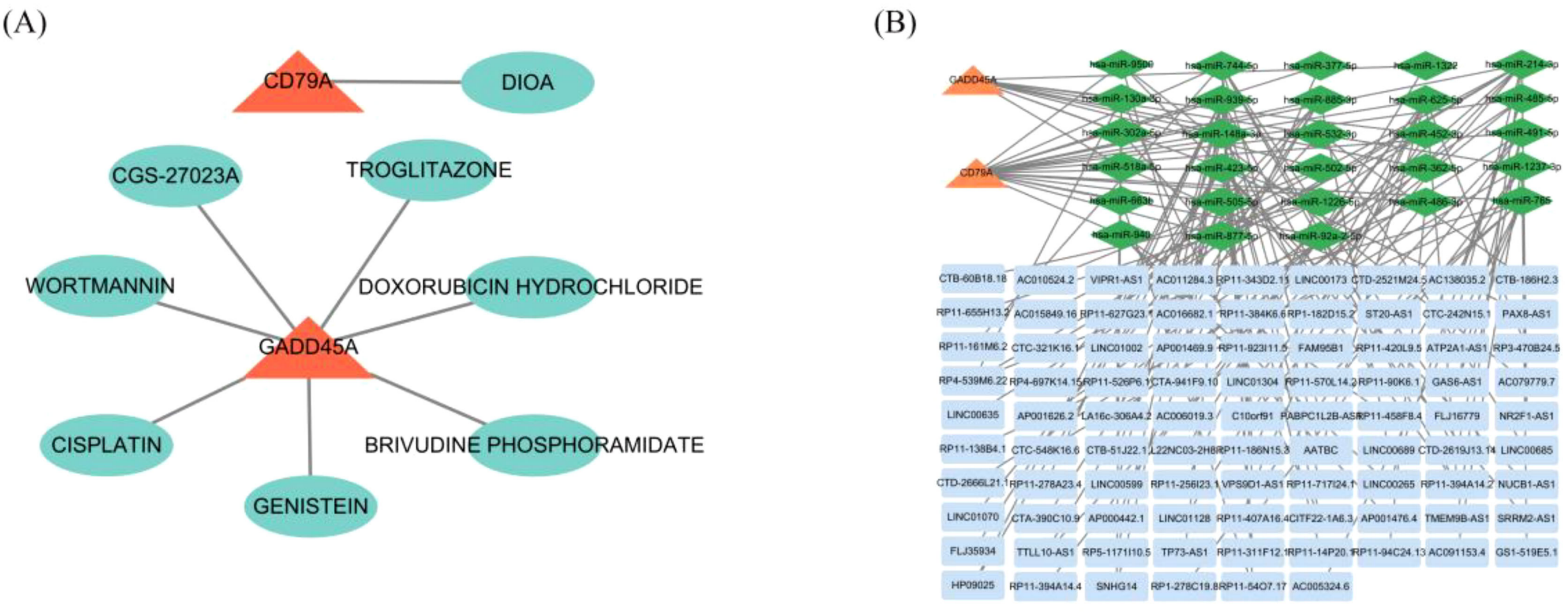
Drug targets and ceRNA network for core genes. **(A)** Predicted drug targets for CD79A and GADD45A using the DGIdb database. **(B)** ceRNA network for CD79A and GADD45A, including mRNAs, miRNAs, and lncRNAs.

We predicted potential drug targets for the hub genes using the DGIdb database. For CD79A, we identified a clear drug target inhibitor, DIOA. For GADD45A, we predicted 7 drugs, including BRIVUDINE PHOSPHORAMIDATE, GENISTEIN, CISPLATIN, DOXORUBICIN HYDROCHLORIDE,CGS-27023A, WORTMANNIN, and TROGLITAZONE (**Figure 7A**). Furthermore, we constructed a ceRNA network consisting of 2 hub gene mRNAs, 28 miRNAs, and 87 lncRNAs (**Figure 7B**), revealing potential regulatory mechanisms of these genes in RSV infection and providing targets for future therapeutic strategies.

### 3.7 Validation analysis

The clinical baseline characteristics of severe and non-severe RSV-infected children are summarized in **Table 1**. Among the 30 severe cases and 62 non-severe cases, no significant differences were observed in gender distribution (male: 60.0% vs. 62.9%, (p = 0.788), term birth status (90.0% vs. 91.9%, (p = 0.757), dwelling environment (63.3% rural vs. 50.0% urban, (p = 0.229), or presence of background diseases (13.3% vs. 1.6%,(p = 0.067). However, severe cases exhibited significantly younger age (median age: 0.4 years [IQR: 0.3–0.8] vs. 0.9 years [IQR: 0.6–2.0], (p < 0.0001), lower hospitalized weight (median: 7.35 kg [IQR: 5.5–9.0] vs. 9.0 kg [IQR: 7.5–11.7], (p < 0.0001), and longer hospital stays (median: 8.0 days [IQR: 7.0–10.3] vs. 6.0 days [IQR: 5.0–6.0],(p < 0.0001)). Additionally, severe cases had a higher incidence of panting (46.7% vs. 14.5%,(p = 0.001), but no differences were observed in fever or elevated C-reactive protein levels. These findings highlight age, weight, and respiratory distress (panting) as critical clinical indicators of RSV severity.

### 3.8 Real-time PCR validation of CD79A and GADD45A expression levels

**Figure 8:**
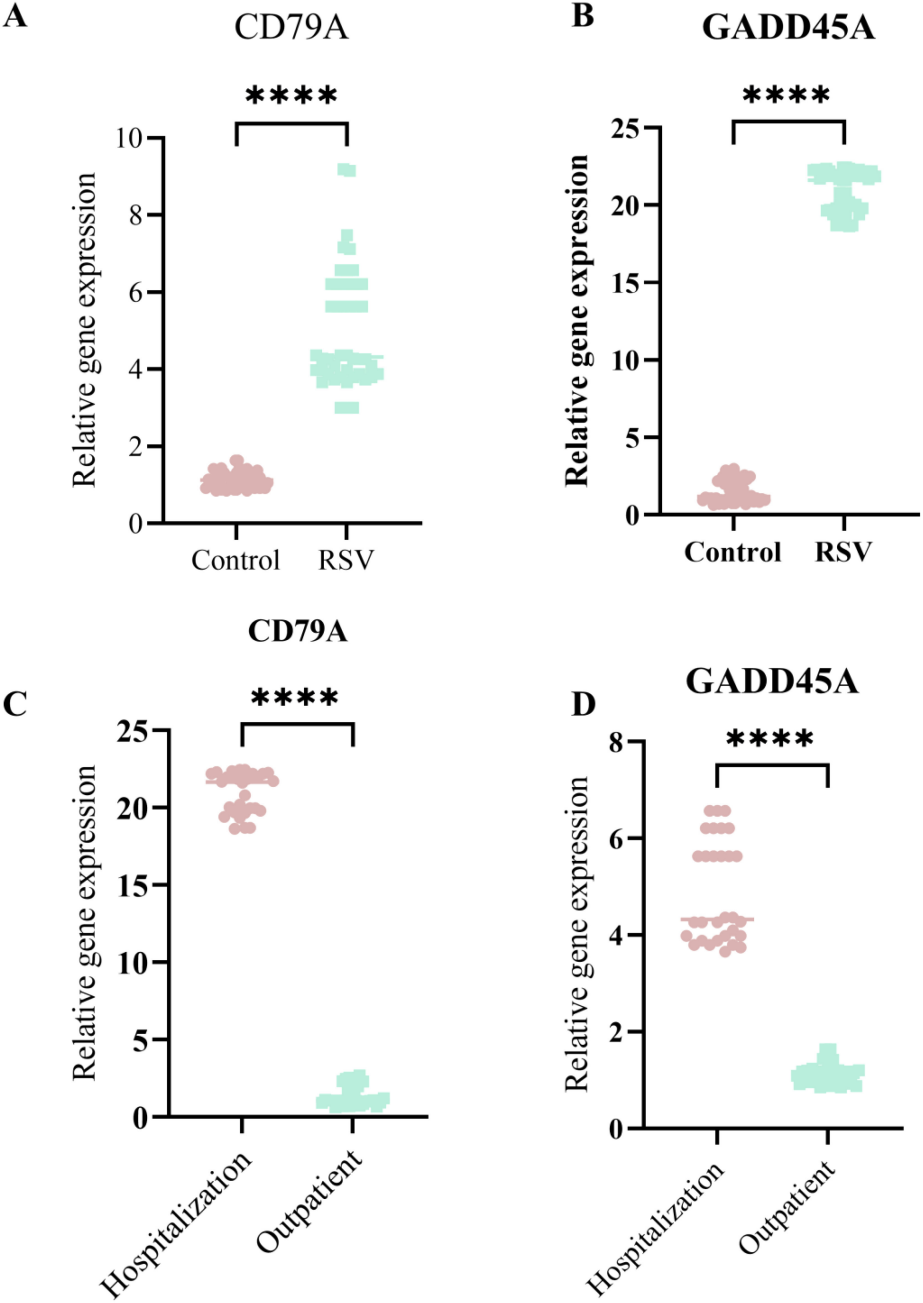
The qPCR validation CD79A and GADD45A expression levels in sever RSV infection children and no sever children. **(A)** CD79A expression significantly higher in RSV group than Control. **(B)** GADD45A expression significantly higher in RSV group. **(C)** CD79Aexpression higher in Hospitalization than Outpatient. **(D)** GADD45A expression higher in Hospitalization. Asterisks indicate statistical significance. ****p<0.0001.

Real-time PCR analysis confirmed that CD79A expression was significantly down-regulated in RSV-infected children compared to healthy controls (p < 0.001). In contrast, GADD45A expression was markedly upregulated in RSV-infected children, particularly in severe (inpatient) cases (p < 0.001). And CD79A and GADD45A expression levels are significant up-regulation in sever RSV infected children (p < 0.001) (**Figures 8A–D**).

## 4 Discussion

The present study identified CD79A and GADD45A as novel biomarkers capable of distinguishing severe from non-severe RSV infections in children and young children. Although a relatively lenient threshold of |log2FC| > 0 was used initially, supplementary analyses confirmed that the key genes (e.g., CD79A, GADD45A) remained significant when applying a stricter |log2FC| > 1 (FDR < 0.05) criterion. Considering the sample size and cross-platform variability, we opted for a sensitive initial threshold to avoid omitting biologically relevant genes with modest fold changes. This strategy was complemented by machine learning-based prioritization to enhance biological relevance and predictive utility. Integration analysis of transcriptomic datasets, machine learning-based feature selection, and clinical validation, these genes demonstrated significant differential expression patterns and diagnostic utility in predicting hospitalization needs. The findings provide critical insights into the molecular mechanisms underlying RSV severity and highlight the interplay between host immune responses and viral pathogenesis. Below, we contextualize these results within the broader understanding of RSV immunobiology, discuss their biological plausibility, and explore their implications for understanding disease progression.

CD79A, a component of the B-cell receptor (BCR) complex, plays a pivotal role in B-cell development and antigen presentation (13). Its down-regulation in RSV-infected inpatients aligns with previous observations of impaired B-cell function during severe viral infections. Reduced CD79A expression may reflect B-cell exhaustion or dysregulated humoral immunity (14), limiting the production of neutralizing antibodies and exacerbating RSV-induced pathology. This hypothesis is further supported by immune cell infiltration analysis, which revealed lower levels of memory B cells and activated plasma cells in hospitalized children. These cell types are critical for mounting adaptive immune responses, and their suppression could prolong viral persistence, increase inflammation, and necessitate prolonged hospitalization. The positive correlation between CD79A expression and activated NK cells or CD8+ T cells further underscores its role in coordinating cytotoxic immune responses, which are essential for viral clearance (15–17).Conversely, its negative association with M0 macrophages and neutrophils suggests that CD79A downregulation may contribute to unresolved inflammation, a hallmark of severe RSV infections.

GADD45A, a stress-response gene involved in DNA repair and apoptosis (18), exhibited marked upregulation in hospitalized RSV patients. This finding aligns with studies linking GADD45A overexpression to cellular damage caused by viral replication. RSV induces oxidative stress and endoplasmic reticulum (ER) disturbances in infected epithelial cells, triggering pathways such as the unfolded protein response (UPR) (19). The GO and KEGG analyses in this study highlighted ER stress and protein folding as enriched pathways among differentially expressed genes (DEGs), providing a plausible mechanistic link between GADD45A activation and RSV severity. Furthermore, GADD45A’s positive correlation with neutrophils and M0 macrophages cell types associated with tissue damage and pro-inflammatory cytokine production suggests its involvement in amplifying inflammatory cascades. Neutrophil extracellular trap (NET) formation, another enriched pathway, is known to exacerbate lung injury in severe RSV cases. Funchal GA et al. research RSV fusion protein promotes human neutrophil extracellular trap formation through a Toll-like receptor 4-dependent mechanism and exacerbate inflammatory symptoms in young children and children (20). Moreover, Cortjens B.et al revealed that Neutrophil extracellular traps cause airway obstruction during respiratory syncytial virus disease (21).Compared with the healthy people, the expression levels of GADD45a were up-regulated in RSV infected and the hospitalized RSV patients, these indicated that GADD45A may drive NET formation in RSV infected patients and leading to the disease progression.

The role of GADD45A and CD79A have the significant regulation for inflammation and the cell stress response, and these biological process main parts are immune cells. Herein, we further explored the association immune cell infiltration and severity RSV patients. The immune cell infiltration profiles revealed distinct patterns between outpatient and inpatient cohorts. Hospitalized children exhibited higher proportions of naïve B cells and M0 macrophages, indicative of an immature or suppressed immune state. Naïve B cells require activation to differentiate into antibody-producing plasma cells or memory cells, and their predominance in severe cases may reflect a failure to transition to adaptive immunity.M0 macrophages, which can polarize into pro-inflammatory M1 or anti-inflammatory M2 subtypes, were more abundant in inpatients, suggesting unresolved polarization that perpetuates inflammation. In contrast, outpatient profiles featured elevated memory B cells, activated NK cells, and M1 macrophages, all of which are associated with effective viral containment. These findings align with previous reports that robust NK cell activity correlate with milder RSV outcomes (22). Enhanced innate immune activation induces protective RSV-specific lung-resident memory T cells in neonatal mice (23). The inverse relationship between GADD45A expression and M1 macrophages further supports the notion that cellular stress pathways inhibit protective immune responses, creating a permissive environment for viral proliferation, the same results can be found in the An L et al. Study showed that Qingdian Oral Liquid promotes fatty acid-dependent M1 to M2 macrophage polarization via the Akt signaling pathway, thereby alleviating RSV-induced lung inflammation (24).

All of the biological process may be regulated by various moleculars, and these molecular regulated by each other compose the pathways. In order to learn these DEGs’ as the parts of what signaling pathways and their biological functions. The 81 overlapping DEGs enrichment functions in pathways related to ER stress, viral genome replication, and hydrogen peroxide catabolism. ER stress is a well-documented consequence of RSV infection, as viral proteins overload the ER’s protein-folding capacity, activating the UPR (25). Persistent ER stress triggers apoptosis and inflammatory signaling, which may explain the prolonged hospitalization observed in severe cases (19). The association of DEGs with hydrogen peroxide catabolism underscores the role of oxidative stress in RSV pathology. RSV infection generates reactive oxygen species (ROS), which damage host cells and promote viral replication (26). GADD45A’s involvement in oxidative stress responses may exacerbate this cycle, leading to tissue damage and clinical deterioration. Additionally, the enrichment of pathways like ‘Natural killer cell mediated cytotoxicity’ and ‘Apoptosis’ highlights the dual role of immune activation-protective in mild cases but destructive when dysregulated in severe infections.

The ROC curve analyses demonstrated that CD79A and GADD45A expression levels effectively differentiated inpatient from outpatient children, with AUC values of 0.758 and 0.716, respectively. While these values indicate moderate diagnostic accuracy, their combination with clinical parameters (e.g., age, weight, and C-reactive protein levels) could enhance predictive power. CD79A’s down-regulation in severe cases may serve as an early warning sign of inadequate B-cell responses, whereas GADD45A up-regulation could signal escalating cellular stress. These biomarkers complement existing clinical criteria, offering a molecular basis for triaging RSV patients.

Previous studies have identified immune-related genes, such as IFNs and chemokines, as potential biomarkers for RSV severity (27).However, the novelty of CD79A and GADD45A lies in their specific association with hospitalization needs rather than general infection status. For instance, while IFN-γis recognized as a key antiviral cytokine in RSV, its suppression in lung epithelia limits its diagnostic utility. In contrast, CD79A and GADD45A reflect broader immune and cellular stress pathways, making them more robust indicators of disease progression. The correlation between CD79A and B-cell subsets aligns with reports of B-cell depletion in severe RSV, while GADD45A’s role in oxidative stress mirrors findings in other viral infections, such as Epstein-Barr virus and SARS-CoV-2 (28, 29). This study has several limitations. First, the analysis relied on blood transcriptomes, which may not fully capture tissue-specific responses in the respiratory tract. Second, the sample size, though adequate for machine learning models, requires expansion to validate generalizability. Third, the functional roles of CD79A and GADD45A in RSV pathogenesis remain hypothetical; mechanistic studies *in vitro* or in animal models are needed to establish causality. Finally, Some patients with RSV infection were co-infected with other viral or bacterial pathogens, which may contribute to the complexity of assessing disease severity in RSV patients. These co-infections could introduce additional molecular markers that confound the evaluation of RSV-specific severity. However, in this study, we specifically included only cases with single RSV infections, both in patients with mild and severe RSV infections, to minimize this confounding effect. Due to the small sample size, further follow-up studies with larger cohorts are needed to validate our findings and better understand the impact of co-infections on RSV severity assessment.

## 5 Conclusion

In summary, this study identified CD79A and GADD45A as potential biomarkers for assessing the severity of RSV infection. Their expression patterns reflect critical aspects of host-pathogen interactions, including B cell dysfunction, oxidative stress, and endoplasmic reticulum (ER) dysfunction. By integrating transcriptomic data with clinical parameters, these biomarkers provide a robust framework for stratifying RSV patients and facilitating the development of personalized intervention strategies. For patient risk stratification, measuring the expression levels of CD79A and GADD45A enables clinicians to more accurately predict the severity of RSV infection and to identify high-risk patients who are more likely to experience severe outcomes. In guiding treatment decisions, these biomarkers help determine the need for hospitalization, the appropriate intensity of supportive care, and the potential use of antiviral or anti-inflammatory therapies. For monitoring disease progression, continuous assessment of CD79A and GADD45A expression may provide insights into the clinical course and treatment efficacy. Future studies should focus on elucidating the precise molecular mechanisms through which CD79A and GADD45A influence RSV pathophysiology.

## Data Availability

The datasets presented in this study can be found in online repositories. The names of the repository/repositories and accession number(s) can be found in the article/supplementary material.
